# Computational drug repurposing study elucidating simultaneous inhibition of entry and replication of novel corona virus by Grazoprevir

**DOI:** 10.1038/s41598-021-86712-2

**Published:** 2021-03-31

**Authors:** Santosh Kumar Behera, Nazmina Vhora, Darshan Contractor, Amit Shard, Dinesh Kumar, Kiran Kalia, Alok Jain

**Affiliations:** 1 Department of Biotechnology, National Institute of Pharmaceutical Education and Research (NIPER) – Ahmedabad, Palaj, Gandhinagar, 382355 Gujarat India; 2 Department of Medicinal Chemistry, National Institute of Pharmaceutical Education and Research (NIPER) – Ahmedabad, Palaj, Gandhinagar, 382355 Gujarat India; 3 Present Address: Department of Bioengineering, Birla Institute of Technology, Mesra, Ranchi, 835215 India

**Keywords:** Computational biophysics, Drug discovery and development

## Abstract

Outcomes of various clinical studies for the coronavirus disease 2019 (COVID-19) treatment indicated that the drug acts via inhibition of multiple pathways (targets) is likely to be more successful and promising. Keeping this hypothesis intact, the present study describes for the first-time, Grazoprevir, an FDA approved anti-viral drug primarily approved for Hepatitis C Virus (HCV), mediated multiple pathway control via synergistic inhibition of viral entry targeting host cell Angiotensin-Converting Enzyme 2 (ACE-2)/transmembrane serine protease 2 (TMPRSS2) and viral replication targeting RNA-dependent RNA polymerase (RdRP). Molecular modeling followed by in-depth structural analysis clearly demonstrated that Grazoprevir interacts with the key residues of these targets. Futher, Molecular Dynamics (MD) simulations showed stability and burial of key residues after the complex formation. Finally, Molecular Mechanics Poisson-Boltzmann Surface Area (MM-PBSA) analysis identified the governing force of drug-receptor interactions and stability. Thus, we believe that Grazoprevir could be an effective therapeutics for the treatment of the COVID-19 pandemic with a promise of unlikely drug resistance owing to multiple inhibitions of eukaryotic and viral proteins, thus warrants further clinical studies.

## Introduction

The global COVID-19 outbreak, by SARS-CoV-2 (2019nCoV), has put the world on a standstill owing to its cross-contamination (transmission)^1^. As per the WHO COVID-19 dashboard (15^th^ Jan. 2021), globally, 90,335,008 confirmed cases had been reported with 1,954,336 associated deaths^2^. Additionally, people’s lives around the globe have suffered as a consequence of compulsory quarantines/isolations or restrictions. Such a critical and pandemic situation demands immediate therapeutic rescue; however, no proven therapy has been established so far^3^.This causes a shift in the policy “from drug discovery to drug repurposing” and, in this course, in vitro or in silico drug screening tool identified few clinically proven FDA-approved drugs as a possible treatment for COVID-19^4,5^. Towards this end, anti-viral drugs like remdesivir (RdRP inhibitor), favilavir (RdRP inhibitor), lopinavir (protease inhibitors), ritonavir (protease inhibitors), and antimalarial drugs until recently (chloroquine and hydroxychloroquine) are top candidates presently being repurposed and under clinical trials for the treatment of SARS-CoV-2^6^.

SARS-CoV-2 belongs to the subfamily orthocoronavirinae, is relatively large, having helical symmetry nucleocapsid with a positive-sense single-stranded RNA genome^7^. The infection begins with the viral entry into the host cell following the ligation of viral spike glycoproteins (S glycoprotein) through the receptor-binding domain (RBD) to the cellular protein (receptor) angiotensin-converting enzyme 2 (ACE2) primed by an enzyme called TMPRSS2 (transmembrane serine protease 2)^8,9^. Once SARS-CoV-2 reaches the host cell, it uncoats to complete the genome transcription and following the translation process. The genome replication and transcription occur at cytoplasmic membranes, mediated by the viral replicase, a protein complex encoded by the 20-kb replicase gene. The replicase complex is comprised of 16 viral subunits and a number of cellular proteins (enzymes), including RNA-dependent RNA polymerase (RdRP), RNA helicase, and protease. The final protein packing takes place at the cell membrane, and the mature virus is formed by budding from the internal cell membranes^10,11^.

Targeting two or more disease pathways is the mainstay of current therapy, which typically relies on combination of two or more separate therapies^12^. However, optimization of the best combinations is generally time-consuming and expensive in the clinical development^13^. In order to streamline combination dosing regimens, the development of molecules with dual or multiple inhibitions capabilities against two or more different classes of the target would be ideal^14^. Given that ACE-2 / TMPRSS2 (eukaryotic) are critical for the fusion of SARS-CoV-2 with host cells and RdRP (viral protein) is essential for viral RNA synthesis, the simultaneous inhibitions of these enzymatic targets by a single-molecule could be a compelling strategy for the treatment of COVID-19^8^. Here we would like to mention that there are several other viral proteins that could be the target. However, it is well established that in the case of COVID-19, at the time of replication, the positive and negative–strands complex by means of RdRP, which is then used for further rounds of translation, RNA replication, and ultimately packaging into progeny virions. Thus, it serves as a hot target to terminate viral replication.

To the best of our knowledge, the approach of targeting eukaryotic host cell proteins and viral protein via a single molecule is an unexplored approach for COVID-19 therapy. We believe this strategy will be more effective considering the analysis of the results of various ongoing clinical trials (single drug therapy vs. combinatorial therapy) and offers unlikeness of drug resistance via viral mutation owing to multiple targets inhibition of differential origin. Although naturally occurring molecules like δ-viniferin, myricitrin, nympholide A, afzelin, biorobin, hesperidin, and phyllaemblicin B exhibited a strong binding to SARS-CoV-2 main protease (M^Pro^)^15^, as well as other targets protein like ACE-2 and RdRP, their direct utilization for COVID-treatment, seems poor as these molecules lack detailed studies including safety profile before getting FDA approval.

Multi-target directed ligands (MTDL’s) are the drugs with two or more pharmacophores, which are structurally overlapping, or separated by a spacer, in a single molecule^16^. The use of computational tools revolutionized this approach by predicting the association of ligand(s) with respective receptors. It becomes more relevant if clinically proven FDA approved drugs are considered for the studies^17^. In this context, in a quest to find similar MTDL(s), we ventured into several anti-viral drugs already in the market and reported here the computational drug repurposing study for their binding affinity with ACE 2 (host cell), TMPRSS2 (host cell), and RdRP (viral) proteins. Grazoprevir (Figure S1), an azamacrocyclic compound indicated for hepatitis C, appeared to be possessing an optimal binding affinity for these three key proteins. The molecular dynamics (MD) simulations and conformational analysis predicted the stable interactions of Grazoprevir with ACE2, TMPRSS2, and RdRP proteins with a promise of the successful therapeutic intervention for COVID-19. Yet these interactions are purely non-covalent and transient in nature. Molecular Mechanics-Poisson Boltzmann Surface Area (MM-PBSA) analysis revealed that van der Waals (vdW) energy have major contributions in binding free energies for all the complexes that makes them transiently stable. This may ensure the drug’s activity based on its postulating or eliminating from the site of interaction. Thus, the chances of toxicity to host cells will be lesser. We strongly believe that our approach for identification of multi-target-directed United States Food and Drug Administration (USFDA) approved drug Grazoprevir may provide new avenues for the therapeutic management of SARS-CoV-2 infection.

## Results and discussions

### Molecular docking study: Grazoprevir interacts with the key residues of ACE2, TMPRSS2, and RdRP

Molecular docking describes the “best-fit” orientation of a ligand to a particular protein. This is fundamentally an optimization problem, which is of high interest in computational studies^18^. To unzip the potential anti-COVID activity among selected 45 ligands (FDA approved anti-viral drugs), molecular interaction along with its conformations in the binding site of the key targeted proteins ACE2 (host cell protein), TMPRSS2 (host cell protein), and RdRP (viral protein), the docking analysis was performed using the Autodock as discussed in the method section^19^.

All the docked conformation of each ligand was ranked according to its binding energy from highest to lowest (see, supplementary Table 1). All the conformations were visualized and analyzed. Among 45 ligands, the top ten ligands were selected for further analysis (Table 1).

**Table 1:** Binding Energy of top ten ligands to the respective protein.

S. no	Drugs	BE (Kcal/mol) (ACE-2)	Drugs	BE (Kcal/mol) (TMPRSS2)	Drugs	BE (Kcal/mol)(RdRP)
1	Paritaprevir	− 7.5	Paritaprevir	− 12.18	Paritaprevir	− 9.59
2	Rilpivirine	− 7.42	Asunaprevir	− 10.55	Grazoprevir	− 8.99
3	Saquinavir	− 7.24	Grazoprevir	− 10.15	Rilpivirine	− 8.16
4	Doravirine	− 6.96	Nelfinavir	− 8.94	Tipranavir	− 8.14
5	Pleconaril	− 6.5	Delavirdine	− 8.72	Rimantadine	− 7.73
6	Grazoprevir	− 6.32	Etravirine	− 8.18	Delavirdine	− 7.66
7	Efavirenz	− 6.3	Saquinavir	− 8.15	Asunaprevir	− 7.35
8	Tipranavir	− 6.22	Indinavir	− 8.05	Etravirine	− 7.12
9	Asunaprevir	− 6.18	Amprenavir	− 8.03	Pleconaril	− 7.01
10	Delavirdine	− 6.17	Boceprevir	− 7.68	Boceprevir	− 6.88

**Figure 1:**
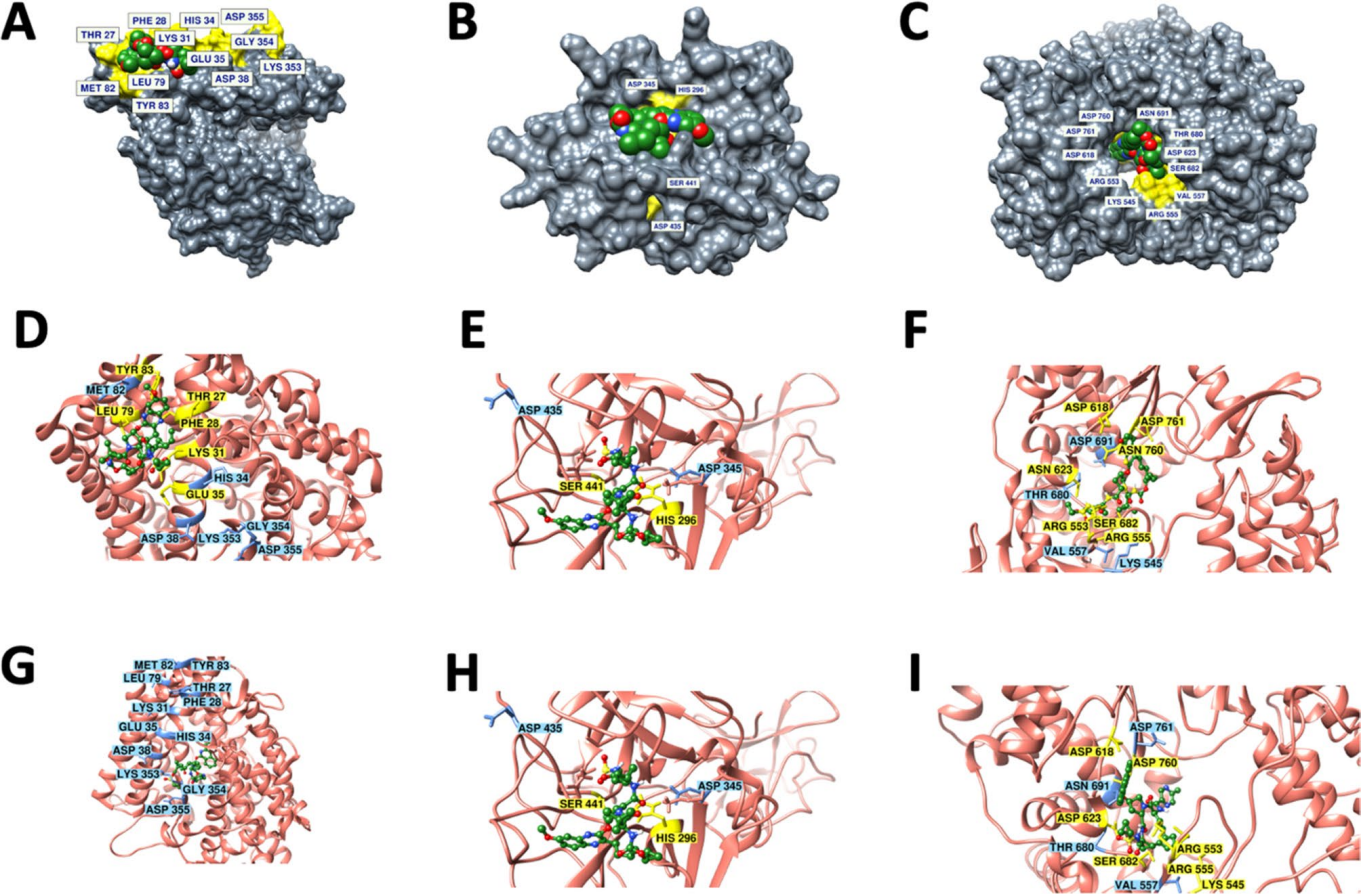
Surface plot and 2D interaction map of protein–ligand interactions of (**A**, **D**) ACE2-Grazoprevir (**B**, **E**) TMPRSS2-Grazoprevir and (**C**, **F**) RdRP-Grazoprevir complexes. (**G**) ACE2-Paritaprevir (**H**) TMPRSS2-Paritaprevir (**I**) RdRP-Paritaprevir complexes showing best fit conformation having the highest binding energy selected for each protein, for our further studies, out of all the conformations generated. Grazoprevir is shown in space filled representation in the the top pannel. Key residues that are reported to be critical either for SARS-CoV-2-host interactions and/or the catalytic activity of these proteins are displayed in yellow and the ones not interacting yet important are displayed in blue. Proteins and ligands are shown in cartoon and ball & stick representations respectively in middle and lower pannel.

Paritaprevir and Grazoprevir were among the top ten ligands which reflected an optimal binding affinity with all the three target proteins. This is worth mentioning, that despite better binding affinity of Paritaprevir, Grazoprevir was selected for further analysis as it interacts with the key residues of the target protein, particularly ACE-2 and RdRP (Fig. 1 and Figure S2) as discussed below. In case of ACE2, Grazoprevir interacts with the residue Gln76, forming a H-bond while residues Thr27, Phe28, Lys31, Glu35, Leu39, Phe72, Glu75, Leu79 and Tyr83 are involved in hydrophobic/polar interactions as displayed in Fig. 1D and Figure S2A. On the other hand, out of all the conformations generated for Paritaprevir, none was shown to bind with the critical residues of ACE-2 (Fig. 1G and Figure S2D). In the case of RdRP, both Grazoprevir and paritaprevir interact with similar numbers of key residues (Fig. 1 and Figure S2). However, a thorough analysis showed that Grazoprevir is able to interact with key residues Arg553, Arg555, and Asp623 through five H-bonds and exhibits strong H-bond network with residues Arg555 and Arg553 of nucleoside triphosphate (NTP) entry channel in motif F of RdRP^20^ (Figure S2C), whereas paritaprevir could not able to interact with any key residues through H-bonds (Fig. 1I and S2F). It is important to mention that H-bond is one of the important non-covalent interactions responsible for the specific to the ligand–protein complexation and thereof drug action. Therefore, in spite of better binding affinity, paritaprevir may be less potent in inhibiting the NTP entry channel compared to the Grazoprevir; hence was not considered for further evaluation. Further, we would like to mention that in the case of TMPRSS2, both Grazoprevir and paritaprevir interact with identical key residues (Fig. 1E, 1H, and Figure S2), therefore if aim is to only target the TMPRSS2, then paritaprevir could be the alternative choice. Considering these above facts intact, the Grazoprevir was selected over paritaprevir as it has optimal interactions with the key residues in case of all the three targets (ACE2, TMPRSS2 and RdRP) protein that takes part in human and virus interaction^20–22^, thus making an integral component for the proposed anti-COVID activity.

### Molecular-dynamic simulations predict the stable binding of Grazoprevir to target proteins

**Figure 2:**
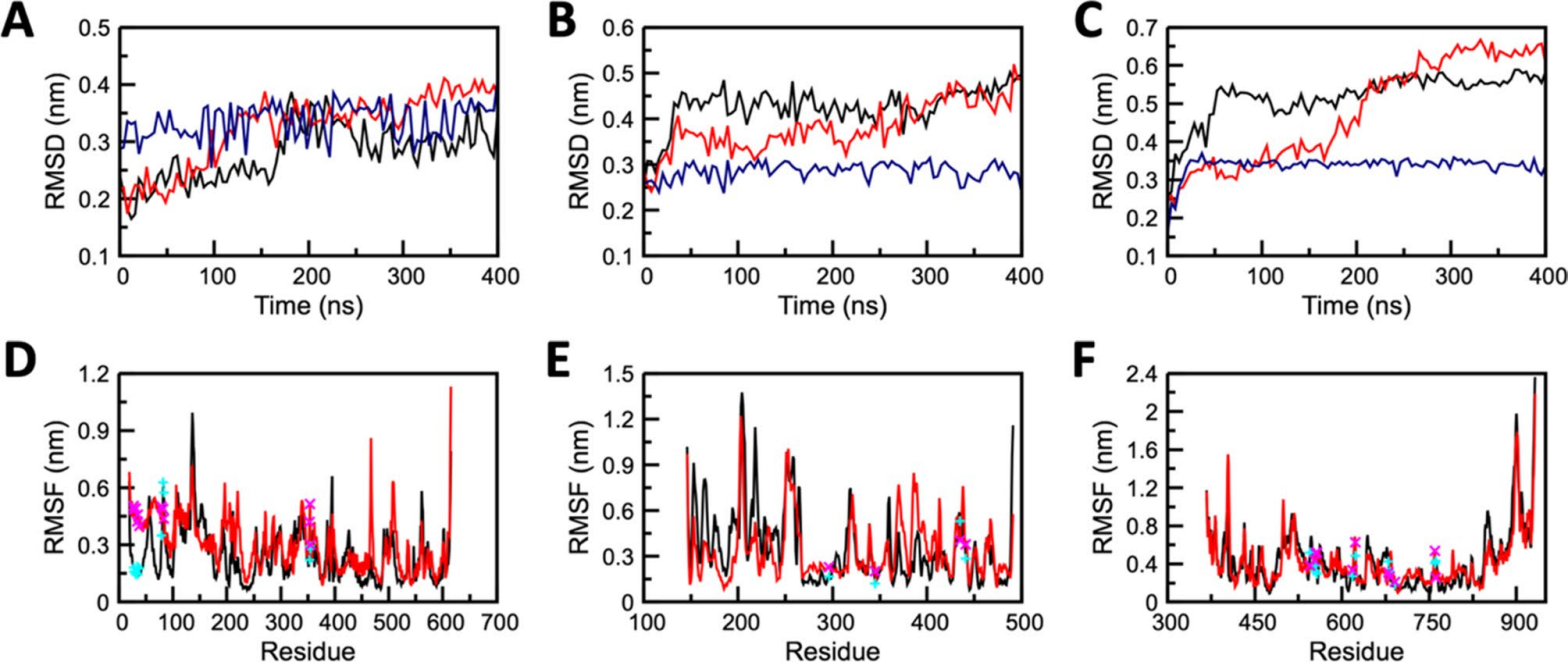
Conformational stability of apo and complex forms of (**A**, **D**) ACE2 (**B**, **E**) TMPRSS2, and (**C**, **F**) RdRP protein. (**A**, **B**, **C**) Backbone-RMSD for protein and all-atom RMSD for Grazoprevir and (**C**, **D**, **E**) Residual RMSF. Profile of apo, complex structures, and Grazoprevir are displayed in red, black, and blue curves , respectively. Locations of key residues are shown by cyan and magenta color symbols for complex and apo states respectively.

Molecular dynamics (MD) is a powerful computational method to predict and analyze the physical movements of atoms and molecules in context of macromolecular structure-to-function relationships. For a specific period of time, the atoms and molecules are allowed to interact, representing the dynamic "evolution" of the system^23^. GROMACS simulation package was used for nanosecond-scale molecular dynamics simulations of Apo (only protein), and Holo (Protein–ligand complex) states of ACE2, TMPRSS2, and RdRP as discussed in the method section. To evaluate system's stability and behaviour in dynamic environment, the backbone Root Mean Square Deviation (RMSD), Root Mean Square Fluctuation (RMSF), Solvent Accesible Surface Area (SASA), intermolecular interactions, Molecular Mechanics Poisson-Boltzmann Surface Area (MM-PBSA), and Principal component analysis (PCA) were measured from the resultant MD trajectories. The dynamic stability of all three systems (repeated twice) was accessed through the RMSD profile of backbone atoms that were plotted for 400 ns (Fig. 2 and Figure S3). The backbone RMSD graph of ACE2-Grazoprevir complex (Holo system), reflected a stable trajectory after 180 ns of simulation (Fig. 2A) when compared to its Apo state. TMPRSS2-Grazoprevir complex exhibits major changes in the first 50 ns in comparison to its Apo state, which later on reached a stable state after 300 ns (Fig. 2B) that is further confirmed by extending the simulations till 500 ns (data not shown). The RdRP-Grazoprevir complex reflected a stable RMSD graph after 60 ns (Fig. 2C), whereas its Apo state had represented non-stable conformations throughout the MD simulations. The Apo systems of all the targets possess a significant structural deviation in comparison with their holo systems. The ACE2-Grazoprevir complex represented a stable RMSD with a value ranging from ~ 0.3 to ~ 0.35 nm, TMPRSS2-Grazoprevir complex represented a stable value ranging from ~ 0.45 to ~ 0.5 nm, whereas RdRP-Grazoprevir complex represented a stable value ranging from ~ 0.5 to ~ 0.55 nm. RMSD values of all the atoms of Grazoprevir were also monitored and displayed as blue curves in Fig. 2A–C. In all the cases, Grazoprevir does not exhibit any significant deviation during the production phase of the simulations that suggest all the rearrangement happened during the equilibration phases (Figure S4) which in turn enhances the binding of Grazoprevir with the receptors as discussed later.

To identify the protein segments/residues contributes to the conformation changes observed during RMSD graph, Root Mean Square Fluctuation (RMSF) of all the systems at the residual level were calculated. The mobility of different residues was observed in all the six systems through RMSF plots (Fig. 2 and Figure S3). Overall higher fluctuations were observed in the Apo forms than Holo forms, which demonstrated the restricted movements throughout the simulation. However, in the Holo state of ACE2, about 10 residues (145–150 and 395–400) exhibited greater deviations in comparison to its apo form. Similarly, in the case of the Holo state of TMPRSS2, about 17 residues (176–188 and 225–230) exhibited greater deviations in comparison its apo form. In the case of RdRP, residues 840–846 exhibited slightly higher deviations in the Holo form in comparison to its apo form. The terminal residues from both the ends of the protein were neglected due to their high mobility. Importantly, the residual RMSF of the key residues displayed relative stable nature compared with the apo state for each of the complexes (Fig. 2). This observation suggests that after the complex formations, key residues were engaged with the ligand that subsequently reduces their fluctutations.

**Figure 3:**
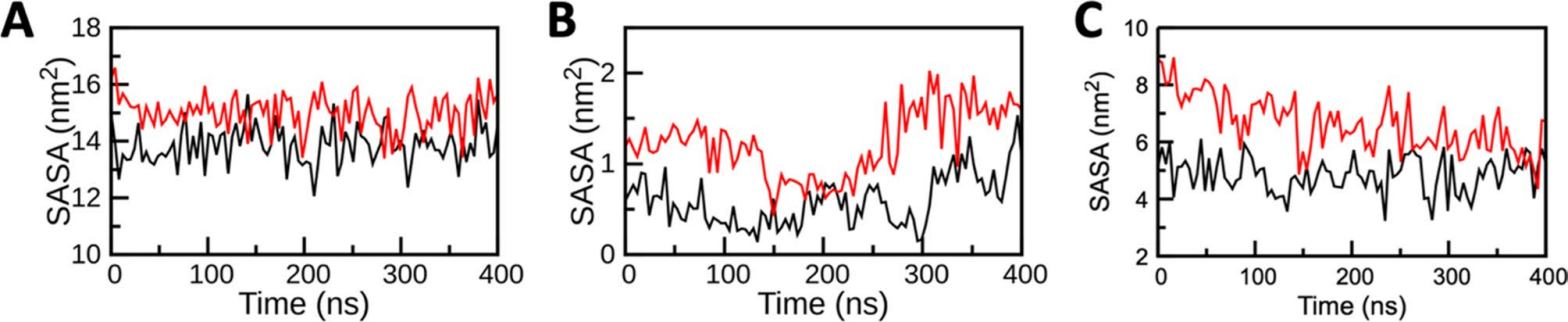
Solvent accessible surface area (SASA) analysis of key residues of ACE2, TMPRSS2, and RdRP during 400 ns MD simulations; (**A**) SASA of ACE2 (**B**)SASA of TMPRSS2and (C) SASA of RdRP. SASA of the complex and apo are displayed by black and red curves, respectively. For ACE2 residues^22^ 24, 27, 28, 30, 31, 34, 35, 37, 38, 41, 42, 79, 82, 83, 330, 353, 354, 355, 357, 393, for TMPRSS2 residues^20,21^ 296, 345, 435, 441 and for RdRP residues^20,21^ 545, 553, 555, 557, 618, 623, 680, 682, 691, 760, 761 were included for this analysis.

Interesting observations from RMSD and RMSF analysis promted us to evalute the solvent accessible surface area (SASA) of the residues that were reported to important for host-virus interaction^22^ or important for the catalytic activity^20,21^. SASA profile (Fig. 3 and Figure S5) clearly indicates the decrease in the SASA of the key residues in the Holo states of ACE2, TMPRSS2, and RdRP when compared with its Apo states. Fewer accessible areas in Holo states reduces the chances of interactions between host and virus and inhibit the enzymatic activities of targeted proteins as key residues are buried in the complex systems. The SASA results specified that there must be some conformational changes in the protein surface and/or it was the effect of comlex formation due to which the amino acid residue shifted from the accessible area to the buried region and lead to the unavailability of key binding/enzymatic residues.

**Figure 4:**
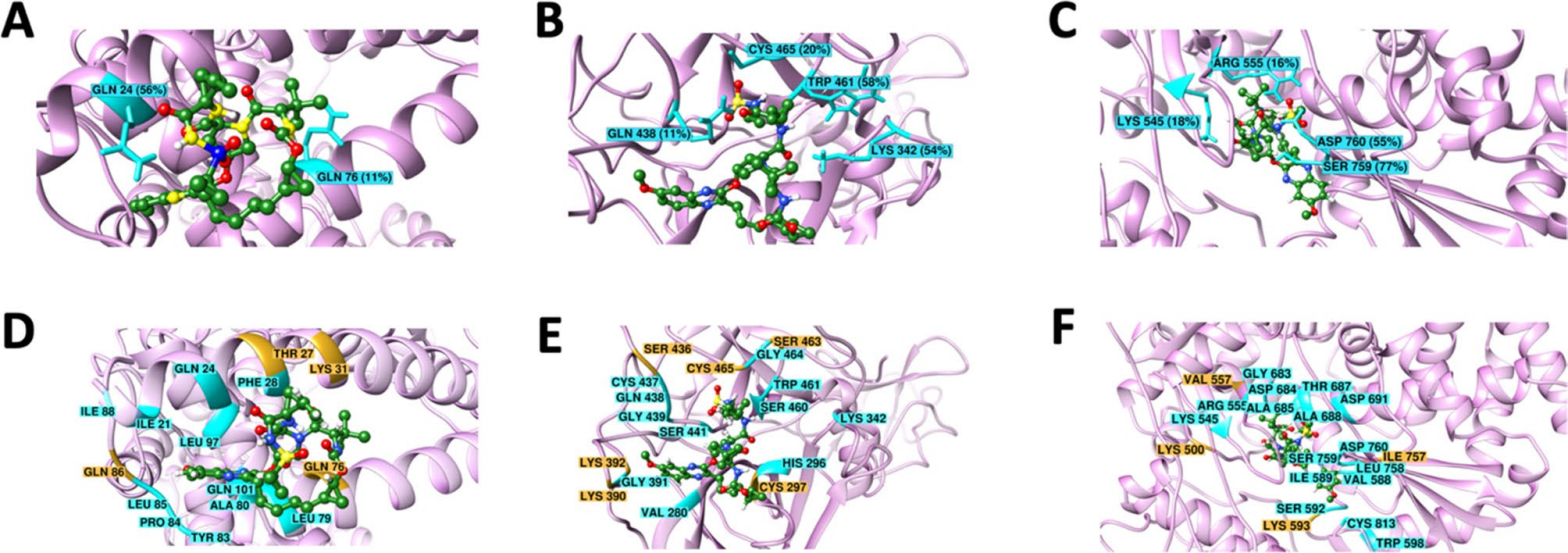
Residues involved in the formation of H-bond (**A**, **B**, **C**) and other non-covalent interactions (**D**, **E**, **F**) between (**A**, **D**) ACE2 and Grazoprevir (**B**, **E**) TMPRSS2 and Grazoprevir (**C**, **F**) RdRP and Grazoprevir. Residues forming H-bond that was stable for more than 10% simulation time are shown by the cyan stick in the ribbon diagram with respective stability. Other stable non-covalent interactions having more than 80% stability are shown by the cyan ribbon. Contacts having 60–80% stability are shown in orange color. Grazoprevir is shown in ball and stick representation, and carbon, oxygen, sulphur and hydrogen atoms are shown in green red, yellow and white.

Generally, the stability of a protein and its complexes is based on the number of H-bonds and intermolecular interactions. In the present context, the total number of hydrogen bonds (intermolecular) was investigated for the Holo states of ACE2, TMPRSS2, and RdRP complex with Grazoprevir, whose results were depicted in Fig. 4 and Figure S6 upper panel. The investigation demonstrated a variable number of intermolecular hydrogen bonds in all the three complexes (repeated twice) during the simulation. A cut-off of > 10% stable H-bonds and > 60% stable contacts were considered for the screening of residues with relatively stable H-bonds and other contacts for the holo states of ACE2, TMPRSS2, and RdRP to rule out the H-bonds that formed only transiently. It is important to mention that only those interactions were classified as a H-bonds if they satisfied both the geometric criteria (r ≤ 0.35 nm or α_HB_ ≤ 30°) otherwise, they were considered as the polar interactions/contacts only. Herein, r is the distance between donor (all the OH and NH groups) and acceptor (all the O and N atoms) and α_HB_ is the angle between hydrogen, donor and acceptor.

Based on the above mentioned cut-off, the ACE2-Grazoprevir complex represented two H-bonds constituting the residues Gln24 and Gln76, which were found to be 56% and 11% stable during the 400 ns simulation time (Fig. 4A). In addition to the H-bond, the residues Ile21, Gln24, Phe28, Leu79, Tyr83, Pro84, Leu85, Ile88, Leu97, and Gln101 formed the contacts, and they were stable for more than 80% of simulation time as depicted in Fig. 4D and S6.In addition to those, other residues namely Thr27, Lys31, Gln76, Gln86 also formed the contact, and their stabilities vary from 60 to 80% simulation time. Subsequently, TMPRSS2-Grazoprevir complex revealed four H-bonds constituting the residues Lys342, Gln438, Trp461and Cys465, which was found to be 56%, 11%, 58%, and 20% stable during the production phase (Fig. 4B and S6). In addition to the H-bond, the residues Val280, His296, Lys342, Gly391, Cys437, Gln438, Gly439, Ser441, Ser460, Trp461, Gly464, were involved in stable contacts with more than 80% stability (Fig. 4E and S6). Other contacts (60–80% stable) were formed by Cys297, Lys390, Lys392, Ser436, Ser463, and Cys465. RdRP-Grazoprevir complex also represented four H-bonds constituting the residues Lys545, Arg555, Ser759, and Asp760, which were found to be 18%, 16%, 78%, and 56% stable (Fig. 4C and S6). Apart from H-bonds, the residues Arg555, Val557, Asp 618, Asp623, Thr680, Ser682, Asn691, Asp760, and Asp761 were involved in other contacts with stability ranging from 60 to 100% during the course of the simulations (Fig. 4F and S6). Majority of these interacting residues were hydrophobic/aromatic, and it can be interpreted that hydrophobic and π-π interactions make the complex more stable. However, contributions of H-bonds and polar interactions cannot be undermined as they provide the specificity of the ligand–protein binding. It is essential to mention that many of the above-mentioned residues namely Gln24, Thr27, Phe28, Lys31, Leu79, Tyr83 for ACE2; H296, S441 for TMPRSS2 and Lys545, Arg555, Ser759 and Asp760 for RdRP are reported to critical for host-virus interaction, catalytic activity and viral replication^20–22^. Interastingly, many of these interactions formed after the rearrangements (includes both orientational and conformational changes) of Grazoprevir during the equilibration phase of the MD simulation. Although, such rearrangement also broken some of initial non-covalent interactions observed during molecular docking but subsequently it fovored to formed many new interactions with other key residues. These interacting residues of targets ACE2, TMPRSS2, and RdRP were crucial in host cell fusion, initiation of viral replication, and transcription cycle of SARS-CoV-2. As they are engaged to form the contacts/interactions with Grazoprevir, they possibly not available to initiate the viral fusion (ACE2), further cleavage (TMPRSS2), and initiate replication (RdRP). Additionally, few of these H-bonds and stable contacts were reported for the first time, which could explore the anti-viral potentiality of Grazoprevir.

To evaluate the energetic contributions of the afore-mentioned non-covalent interactions, end point binding free energy was estimated for all the three protein-Grazoprevir systems. There are ample of methods that have been implemented for the determination of free energy from the MD trajectories^24,25^. Among the various energy terms that contributed to the enzyme–substrate binding energy, van der Waals (E_vdw_), electrostatic (E_ele_), and solvation (E_solvation_) energy played a decisive role to the overall binding free energy and complex stability.

**Figure 5:**
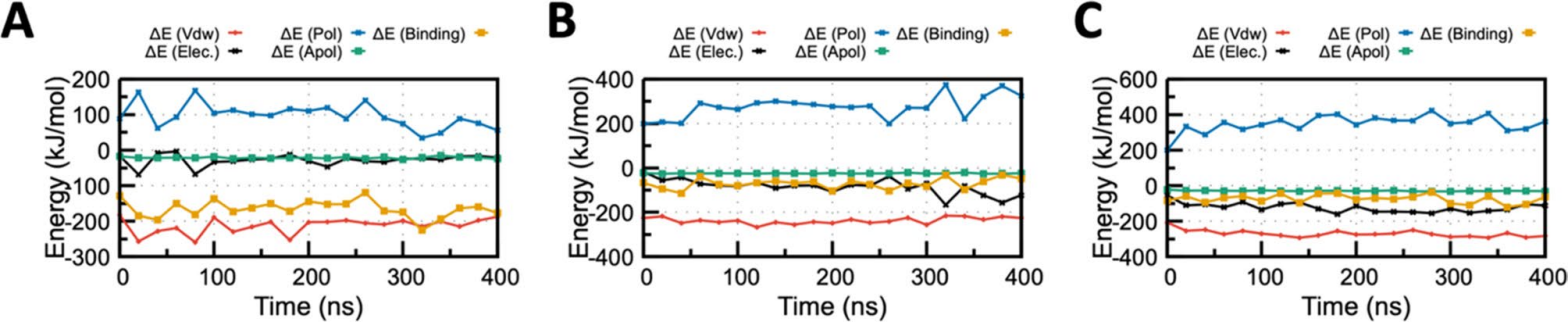
MM/PBSA binding energy profile for the (**A**) ACE2-Grazoprevir (**B**) TMPRSS2-Grazoprevir and (**C**) RdRP-Grazoprevir complex. Contributions from various energy components are also shown for all the three complex. Van der Waals energy (ΔE_vdW_), Electrostatic energy (ΔE_Elec_), Polar solvation energy (ΔE_pol_) , Apolar solvation energy (ΔE_Apol_), Total binding energy (ΔE_Binding_) are displayed by red, black, blue, green and orange curve Figure S7: MM-PBSA binding energy profile for the (**A**) ACE2-Grazoprevir (**B**) TMPRSS2-Grazoprevir and (**C**) RdRP-Grazoprevir complex. Contributions from various energy components are also shown for all the three complex. Van der Waals energy (ΔEvdW), Electrostatic energy (ΔEElec), Polar solvation energy (ΔEpol) , Apolar solvation energy (ΔEApol), Total binding energy (ΔEBinding) are displayed by red, black, blue, green and orange curve respectively.

MM/PBSA, a end point free-energy simulation technique, was implemented, which explored the binding free energy of ACE2, TMPRSS2, and RdRP complexed with Grazoprevir as it is known to have better accuracy compared with docking binding energy^25,26^. The binding free energy decomposition of all the complexes has been summarized in Fig. 5 and S7. The average binding free energy of three complexes (repeated twice), i.e., ACE2, TMPRSS2, and RdRP complexed with Grazoprevir, were analyzed to be − 165.1 ± 5.1, − 71.0 ± 4.8, and − 74.2 ± 5.2 kJ/mol^−1^, respectively. Apart from overall binding free energy, MM/PBSA binding energy of all the drug-target complexes (Grazoprevir complex with ACE2, TMPRSS2, and RdRP) was decomposed to to identify the governing factors responsible for stable complex formation. Electrostatic, van der Waals (vdW), solvation (polar and non-polar), and total binding energy profile of the complex is illustrated in Fig. 5 and S7A. Free energy calculations revealed that vdW energy have a major contribution in binding free energy for all the complexes that make the complex transiently stable. In addition, it was noticed that the contribution of the polar solvation energy was unfavorable in nature. In the ACE2-Grazoprevir complex, the energy contribution was increased from the starting values of − 16 and − 182 kJ/mol to the averaged values of − 28 and − 213 kJ/mol for electrostatic and vdW energies, respectively (Fig. 5A). While in the case of TMPRSS2-Grazoprevir complex, the energy contribution was increased from the starting values of − 17 to − 226 kJ/mol and − 84 to − 237 kJ/mol for electrostatic and vdW, respectively (Fig. 5B). For the RdRP-Grazoprevir complex, the energy contribution was increased from − 55 to − 208 kJ/mol and − 124 to − 270 kJ/mol for electrostatic and vdW, respectively (Fig. 5C). A closer observation identified interesting characteristics of all the three complexes. In the ACE-Grazoprevir complex, a significantly favorable change in the vdW energy term was observed, while for the TMPRSS2-Grazoprevir complex, electrostatic energy changes substantially, and the RdRP-Grazoprevir complex displayed larger perturbation in both electrostatic and vdW energy terms. Such observation nicely correlates with the characteristics of the residues participate in the complex formation as displayed in Fig. 4 and S6. It is important to mention that binding energy trends for Grazoprevir with all the three receptors reversed compared with the docking binding score. Rearrangement of Grazoprevir, and chage in side-chain conformations of interacting residues during MD are the major reasons for it. Additionaly, such discrepancy between docking and MM-PBSA binding energy are frequently observed in many studies because binding free energies measured by docking scores are usually not of high accuracy and has known difficulty in reliably distinguishing compounds with comparable binding affinities^27–29^. Therefore, in the literature binding scores calculated using molecular mechanics combined with the MM-PBSA approach can more accurately predict the binding affinities.

Finally, to infer the mechanical properties like structural motions and fluctuations of all the states of ACE2, TMPRSS2, and RdRP, Principal component analysis (PCA) or Essential dynamics (ED) analysis was carried out. A set of eigenvectors was acquired from the MD trajectories, which depicted the motion of every solitary component through vectorial depictions.

**Figure 6:**
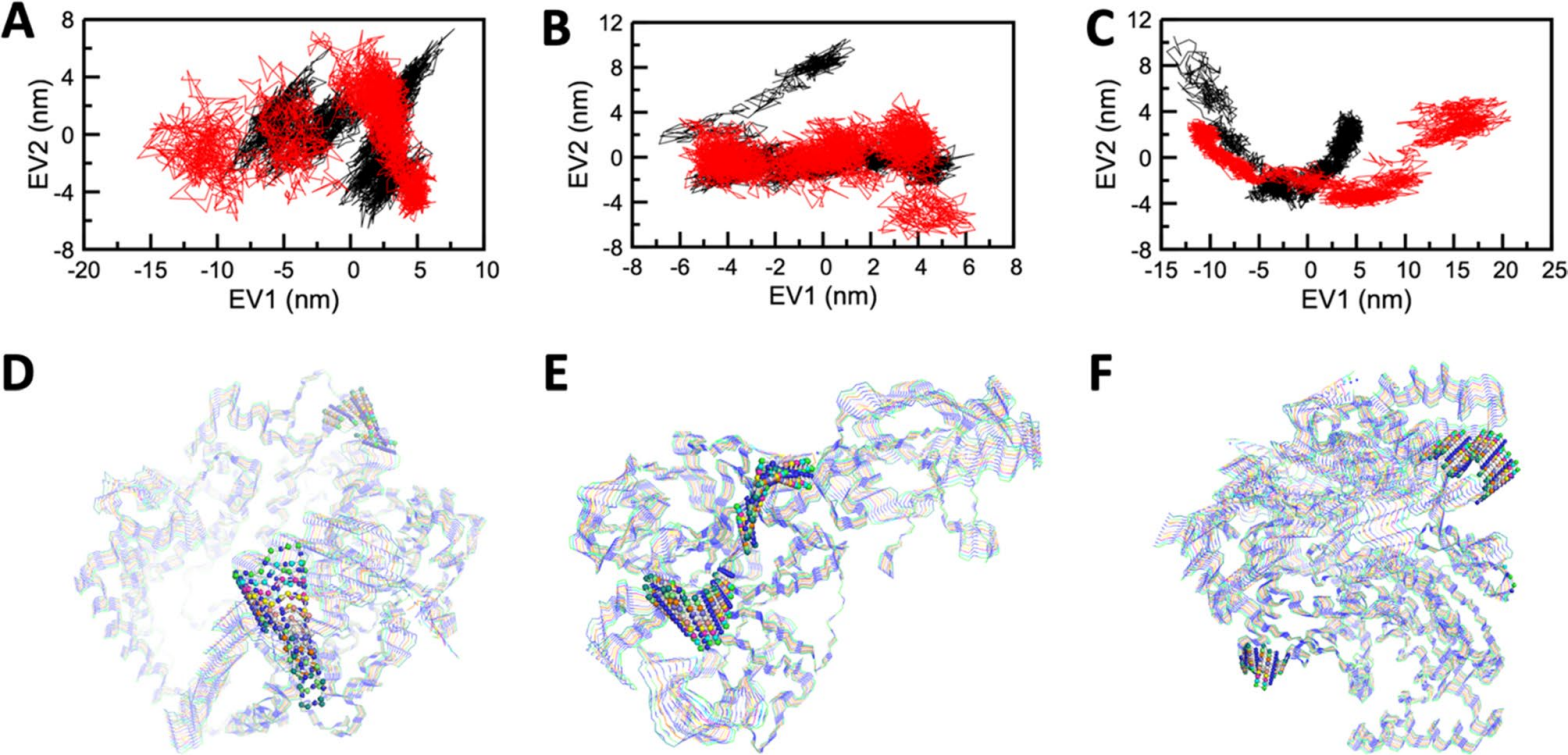
Projection of the motion of the apo and holo forms of ACE2, TMPRSS2, and RdRP in phase space along the first two principal eigenvectors (EV1 and EV2) for (**A**) ACE2 (**B**) TMPRSS2 and (**C**) RdRP. Graphical representation of 10 equally divided structures extracted from dynamic trajectories showing prevalent motions in (**D**) ACE2 (**E**) TMRRSS2 and (**F**) RdRP. Residues found to be highly mobile in comparison to other residues in the targeted proteins depicted in the ball and stick representation.

The structural motions or fluctuations of every component within the Apo and Holo states of ACE2, TMPRSS2, and RdRP protein were illustrated through the first two principal components graphed against EV1 and EV2 (illustrated in Fig. 6 and S8). The graphs represented a higher scattering of components in Apo form in comparison to Holo forms.

Mostly in every simulation protocol, the flexibility of Apo and Holo states were confined and determined by its trace values of the covariance matrix of the backbone atoms. The depicted trace values of Apo and the Holo state of ACE2 was 60.3 nm^2^ and 39.8 nm^2^ (see SI Figure S9 and S10). The trace value of Apo and the Holo state of TMPRSS2 was depicted and was found to be 33.9 nm^2^ and 34.4 nm^2^ (Figure S9 and S10). Similarly, the trace value of Apo and Holo state of RdRP was depicted and was found to be 129.8 nm^2^ and 48.8 nm^2^ (Figure S9 and S10).The lower trace values in both the Holo states confirm the overall decreased in the flexibility of ACE2, RdRP compared to their Apo states, whereas there is no significant change observed for the TMPRSS2-Grazoprevir complex compared to its Apo state. The results of PCA analysis in all the cases of the protein-Grazoprevir complexes were well correlated with its RMSD analysis (Fig. 2 and S3).

The fluctuations or structural motions of the components in the system were well analysed through correlating the graph and trajectory analysis. The dynamic trajectories that were obtained from EV1 of ACE2, TMRRSS2, and RdRP (Fig. 6 and S8) have represented a fluctuation pattern of amino acid residues in each system, which were identified by the superimposition of the 100 conformations with an interval of 10 ns. Most of the residues are seemed to fluctuate, but the residues 133–140, 284–288 in ACE2, 247–253, 256–261 in TMPRSS2, and 642–646, 872–879 in RdRP (represented in the ball and stick model (Fig. 6 and S7 lower panel) were observed to have more mobile regions and may get exposed than others. This depicts that the residues considered in the investigation of SASA were found to have less mobility, which may be due to strong interactions between protein-Grazoprevir complexes. The results of this analysis well aligned with the results of SASA and RMSF profile.

In the current investigation, an integrative approach was performed where the techniques of docking, homology modelling, MD simulations, and MM/PBSA binding free energy techniques were employed. Docking score function was used to predict the binding orientation of ligand and evaluate binding free energy (affinity), followed by MD simulation to obtain the most stable structure of complexes. This approach could explore the residual movements and its key interactions that are not only confined with the intra-molecular but also the intermolecular ACE2, TMPRSS2, and RdRP interactions with Grazoprevir. MM/PBSA binding free energy portrayed that among the various energy terms, van der Waals and electrostatic energy plays a very decisive role to the overall negative binding free energy and stabilities. Furthermore, this study able to unzip the association between the movements of residues in all the targets, Apo, and Holo state trajectories. The results from this investigation could able to signify the potentiality of Grazoprevir for anti-viral therapeutics. Thus, following the multiple computational approaches, we envisage that Grazoprevir could be repurposed for fighting against the COVID-19 pandemic. As predicted, Grazoprevir interacts with all three proteins (host cell as well as virion) optimally with transiently stable binding conformations. Since this drug, Grazoprevir, is marketed as fixed-dose combinations with elbasvir (Zepatier™), which was also reported to have anti-COVID activity recently^30^. Thus, it is encouraging for effective drug combinations to derive their synergistic effects. The propensity of Grazoprevir to bind fundamentally different eukaryotic and SARS-CoV-2 proteins with strong affinities remain unexplained.

## Conclusions

Following multi-target directed ligands (MTDL’s) screening, we disclose Grazoprevir exhibits optimal affinity for the key proteins engaged in viral entry into host cells (ACE2 and TMPRSS2) and its replication (RdRP) assembling new virions as predicted by molecular docking, homology modeling, and molecular simulations (MD) and MM-PBSA studies. The study further warrants the in vitro and in vivo testing before moving forward to the clinical studies and subsequent therapeutic applications (alone or in-combinations). We believe the strategy of targeting host-cell proteins and virion protein simultaneously by a single molecule for COVID-19 therapy will not only improve the patient compliance but also minimize the side effects due to dose reduction, likely drug resistance owing to multiple pathway inhibition, and cost of therapy.

## Material and methods

### Selection of protein, structure retrieval and prediction

Three proteins, namely Angiotensin-Converting Enzyme-2 (ACE2), Transmembrane protease serine 2 (TMPRSS2), and RNA-dependent RNA polymerase (RdRP) (viral protein) were chosen for this investigation. All these proteins play a key role in COVID-19 (SARS-CoV-2 infections) pathophysiology, particularly host cell entry and viral RNA replication. The three-dimensional structure of ACE2 and RdRP was retrieved from Protein Data Bank^31^ (http://www.rcsb.org/pdb/home.do) with PDB ID: 6M0J^22^ and 7BTF.^20^ The co-crystallized Receptor Binding domain (RBD) of spike protein from ACE2 and the co-crystallized cofactors nsp7 and nsp8 of RdRP were separated through BIOVIA Discovery Studio 4.5 Visualizer^32^or UCSF Chimera^33^. The non-interacting ions, all water molecules were removed before the docking. The missing hydrogen atoms were added using UCSF Chimera 1.13, an extensible molecular modelling program^33^.

The three-dimensional structure of TMPRSS2 protein was generated by SWISS-MODEL online server using the amino acid sequence from UniProt (UniProt KB-O15393) due to the non-availability of its crystal structure. Serine protease hepsin’s (PDB: 5CE1) structure was selected as a template which was having the highest sequence identity amongst more than 50 templates available, which was 33.82% using a similar protocol as discussed elsewhere^21,34^. Modelled structure (residues 146–491) included the peptidase S1 domain that is important for its catalytic activity. 92% of residues are within the allowed regions, according to the Ramachandran Plot. The modeled TMPRSS2 protein was cross-validated for its structural coordinates using computational methods.

SARS-CoV-2 RdRP with 942 amino acids was directed for domain search for recognizing the key region of the protein for further analysis. Based on the studies of Gao et al., 2020 it was understood that the structure of the 2019 novel coronavirus (2019-nCoV) nsp12 is composed of a “right hand” RdRP domain (residues S367-F920 ). This RdRP domain constitutes a well-conserved polymerase domain having three subdomains, namely fingers subdomain (L366-A581 and K621-G679), a palm subdomain (T582-P620 and T680-Q815), and a thumb subdomain (H816-E920). Considering the above statements, the RdRP protein with residues from 367 to 932 was selected for further computational analysis.

The binding site of the ACE2, TMPRSS2, and RdRP were identified by taking together the consensus results of CASTp (Computed Atlas of Surface Topography of Proteins), RBD of spike protein binding site (for ACE2), catalytic site (for TMPRSS2), and remdesivir binding site (for RdRP).

### Ligand selection and preparation

Clinically proven Food and Drug Administration (FDA) approved 45 anti-viral drugs that were considered in the current investigation. The structure data file (sdf) format of all the 45 inhibitors was retrieved from the NCBI PubChem database. This .sdf file was converted into pdb through Online SMILES(Simplified Molecular Input Line Entry System) translator web server (https://cactus.nci.nih.gov/translate/) or Open Babel GUI was used to convert or PDBQT format towards input to AutoDock 4.2 (autodock.scripps.edu/) docking tool^35^.

### Molecular docking

The docking protocol is used for extrapolation of ligand/drug conformation and its orientation within a targeted binding site or active site of a protein. AutoDock is the most widely accepted molecular docking tool that is widely used for the screening of compounds against potential targets. In the current investigation AutoDock, 4.2 was used for molecular docking analysis of ACE2, TMPRSS2, and RdRP protein against all the 45 drugs. AutoDock Tools (ADT), v.1.5 was used for assigning Kollman charges^36^ for protein and Gasteiger(-Marsili)^37^ partial charges for all the 45 ligands. To permit fully-extended conformation of ligand, different grid values were chosen for all the three proteins with a particular dimension space and parameters based on x-centering:, y-centering:, and z-centering. Residues that are involved in binding of spike protein (for ACE2), or catalytic activity (for TMPRSS2 and RdRP) were selected for grid generation as illustrated in Fig. 1. A grid box was observed to cover all the selected residues. The Lamarckian genetic algorithm (LGA) was considered as it is one of the best docking methods available in Autodock. The best resultant docked complexes were ranked based on binding energy and intermolecular interactions between ligand and protein covering most of the residues, as shown in Fig. 1. The best docking complexes from each system were screened from the independent molecular docking for further analysis. Lig-Plot + ^38^, UCSF-Chimera^33^ and Biovia Discovery Studio Visualizerv4.5^32^were used for image generation and protein–ligand interaction analysis. In order to understand the dynamics stability and probable mode of ligand/drug binding in the studied targets (ACE2, TMPRSS2, and RdRP ) and its complexes were subjected to MD simulations.

### Molecular dynamics (MD) simulations

In the current investigation, we had employed the MD simulations for the Apo (without drug/ligand) and Holo (complexed with drugs/ligands) systems of targeted proteins ACE2, TMPRSS2, and RdRP using GROMOS 54A7 force-field^39^ using GROMACS suit (version 2019.4)^40^ in order to understand the dynamic behaviour, mode of binding and inhibitor specificity for all the systems. The targeted protein and protein–ligand complex structures were considered from the final docked structures, as discussed above. Automated Topology Builder (ATB)^41^ was used for the generation of force-field parameters of Grazoprevir. The initial structure was solvated using the extended SPC/E water model^42^.All the systems were immersed in a cubic box of SPC/E water molecules with a minimum distance of 12 Å between the protein surface and the edge of the box. The solvated system was neutralized by adding the counter ions. Energy minimization was performed for releasing the conflicting contacts, using the steepest descent method with a tolerance of 10 kJ mol^−1^. Energy minimized systems were subjected to equilibration phase-I in which all the heavy atoms were position restrained for 2 ns in the NVT ensemble. Further, followed by the secondary phase in the NPT ensemble for 2 ns. All the systems were kept at a constant 300 K in association with the velocity-rescale thermostat^43^ with a coupling constant 0.1 ps. During the 400 ns production run, Parrinello-Rahman coupling algorithm^44^ was used for keeping the pressure constant at 1 bar with a coupling constant of 2 ps. Particle Mesh Ewald method^45^ with a cut-off of 1.4 nm was used for the evaluation of long-range nonbonded interactions in the systems and van der Waals interactions. Periodic boundary conditions were applied in all three (x,y,z) directions. All the bonds length were constrained using the LINCS algorithm^46^. SETTLE algorithm^47^ was used to constrain the geometry of water molecules. The trajectories of MD simulations were analyzed by built-in modules of Gromacs or in-house scripts. Root Mean Square Deviation (RMSD), Root Mean Square Fluctuation (RMSF), Solvent Accessible Surface Area (SASA) of key interacting residues, Principal Component Analysis (PCA), and stability of various non-covalent interactions were analyzed. A sphere of water molecules was used to calculate the SASA^48^ of molecules. The stability of non-covalent interactions was measured by *gmx_hbond*, *gmx_mindist,* and in-house scripts. Contacts were defined if the minimum distance between any atoms of protein residues and ligand was within 0.4 nm. PCA was carried out through the essential dynamics (ED) method using *gmx_covera* and *gmx_aneig* modules of Gromacs simulation suit. A set of eigenvectors and eigenvalues were obtained after diagonalzing and calculating the covariance matrix which reflects concerted motion of the molecules. The *gmx_anaeig* tool was employed to analyze and plot the eigenvectors generated from the MD trajectory. In the present investigation, the first two Principal Components (PCs) i.ePC1 and PC2, which dominate the collective motions in Apo and Holo forms, were considered for further analysis. All the complex simulations were repeated two times with different starting velocities. All 2D plots were generated by the GNU plot for data analysis.

### MM/PBSA binding free energy analysis

The binding free energy of all the three complexes (ACE2, TMPRSS2 and RdRP), was analyzed using g_mmpbsa^49^ tool, which is based on the molecular mechanics/Poisson–Boltzman surface area (MM/PBSA) method and well known for its popularity in estimating the interaction free energies of ligands/drugs with proteins/receptors^50^. The *g_mmpbsa* tool was used to analyze the solvation properties of drugs and targets during MD.

The binding free energy (△*G*_*bind*_) was calculated using the following equation:

1 $$\Delta G_{bind} = \, G_{complex} {-} \, G_{protein} {-} \, G_{ligand} = \Delta E_{MM} + \Delta G_{sol} {-} \, T\Delta S$$

2 $$\Delta E_{MM} = \Delta E_{bonded} + \Delta E_{nonbonded} = \Delta E_{bonded} + \, (\Delta E_{vdw} + \Delta E_{ele} )$$

3 $$\Delta G_{sol} = \Delta G_{polar} + \Delta G_{nonpolar}$$

The Van der Waals (E_*vdw*_) and electrostatic interaction (E_*ele*_) are included under non-bonded interactions (E_*nonbonded*_) whereas the bonded interaction energy (E_bonded_) is comprised of bond, angle, dihedral and improper dihedral energy terms. With the applications of Poisson-Boltzmann (PB) equation and solvent-accessible surface area (SASA), the polar solvation free energy and the non-polar solvation free energy can together describe the solvation (△G_sol_) energy. T△S portrays the shift in conformational entropy pertaining to ligand binding, which is frequently overlooked in practise due to its high computational cost and poor accuracy of prediction^51,52^. Additionaly, the net conformational entropic contribution is often small, and its addition in total binding energy only slightly improve the correlations with the experiement^52–54^.

The free binding energy was therefore measured with the following four components: the contribution of Van der Waals (△*G*_*vdw*_), the contribution of electrostatic (△*G*_*ele*_), the polar desolvation portion (△*G*_*polar*_) and the contribution of non-polar (△*G*_*nonpolar*_).

4 $$\Delta G_{bind} = \Delta G_{vdw} + \Delta G_{ele} + \Delta G_{polar} + \Delta G_{nonpolar}$$

In this investigation, we had retrieved the 20 snapshots from the MD trajectories of all the six complexes, individually, to calculate the binding free energy from 0 to 400 ns with an interval of 20 ns.

## Supplementary Information

Supplementary Information
