# Supplementary material for: Computational drug repurposing study elucidating simultaneous inhibition of entry and replication of novel corona virus by Grazoprevir
Source: Sci Rep. 2021 Mar 31;11:7307. doi: 10.1038/s41598-021-86712-2 (PMC8012565; doi:10.1038/s41598-021-86712-2)
Supplement: Supplementary file 1 — Supplementary Information [file 41598_2021_86712_MOESM1_ESM.pdf]

Computational Drug Repurposing Study Elucidating Simultaneous Inhibition of Entry and Replication of Novel Corona Virus by Grazoprevir

Santosh Kumar Behera,#[a] Nazmina Vhora,#[a] Darshan Contractor,#[a] Amit Shard,*[b] Dinesh Kumar,*[b] Kiran Kalia,*[a] Alok Jain,*[a]

[a] Department of Biotechnology
National Institute of Pharmaceutical Education and Research (NIPER) – Ahmedabad, Palaj, Gandhinagar-382355, Gujarat, India

[b] Department of Medicinal Chemistry
National Institute of Pharmaceutical Education and Research (NIPER) – Ahmedabad, Palaj, Gandhinagar-382355, Gujarat, India

Table S1. Binding Energy of ligand (antiviral drug) to the respective protein.

Sr no.	Drugs	BE (Kcal/mol) (ACE-2)	Drugs	BE (Kcal/mol) (TMPRSS2)	Drugs	BE (Kcal/mol) (RdRP)
1	Paritaprevir	-7.5	Paritaprevir	-12.18	Paritaprevir	-9.59
2	Rilpivirine	-7.42	Asunaprevir	-10.55	**Grazoprevir**	-8.99
3	Saquinavir	-7.24	**Grazoprevir**	-10.15	Rilpivirine	-8.16
4	Doravirine	-6.96	Nelfinavir	-8.94	Tipranavir	-8.14
5	Pleconaril	-6.5	Delavirdine	-8.72	Rimantadine	-7.73
6	**Grazoprevir**	-6.32	Etravirine	-8.18	Delavirdine	-7.66
7	Efavirenz	-6.3	Saquinavir	-8.15	Asunaprevir	-7.35
8	Tipranavir	-6.22	Indinavir	-8.05	Etravirine	-7.12
9	Asunaprevir	-6.18	Amprenavir	-8.03	Pleconaril	-7.01
10	Delavirdine	-6.17	Boceprevir	-7.68	Boceprevir	-6.88
11	Tromantadine	-6.17	Tipranavir	-7.66	Doravirine	-6.57
12	Nitazoxanide	-6.13	Zidovudine	-7.42	Amantadine	-6.54
13	Zidovudine	-5.97	Darunavir	-7.33	Podophyllotoxin	-6.51
14	Abacavir	-5.8	Telaprevir	-7.15	Nelfinavir	-6.42
15	Nevirapine	-5.78	Doravirine	-7.1	Nitazoxanide	-6.35
16	Sofosbuvir	-5.77	Atazanavir	-7.06	Ritonavir	-6.26
17	Idoxuridine	-5.62	Lopinavir	-6.93	Tromantadine	-6.25
18	Rimantadine	-5.57	Podophyllotoxin	-6.91	Nevirapine	-6.23
19	Etravirine	-5.54	Abacavir	-6.83	Abacavir	-6.12
20	Podophyllotoxin	-5.44	Nevirapine	-6.83	Indinavir	-6.09
21	Stavudine	-5.32	Pleconaril	-6.81	Sofosbuvir	-5.8
22	Indinavir	-5.31	Rimantadine	-6.76	Lopinavir	-5.75
23	Darunavir	-5.3	Sofosbuvir	-6.74	Efavirenz	-5.66
24	Tenofovir	-4.94	Penciclovir	-6.61	Idoxuridine	-5.6
25	Boceprevir	-4.93	Ritonavir	-6.46	Zidovudine	-5.49
26	Atazanavir	-4.92	Rilpivirine	-6.33	Didanosine	-5.43
27	Trifluridine	-4.91	Efavirenz	-6.28	Emtricitabine	-5.39
28	Nelfinavir	-4.86	Nitazoxanide	-6.26	Amprenavir	-5.37

29	Didanosine	-4.79	Famciclovir	-6.21	Darunavir	-5.07
30	Lamivudine	-4.74	Stavudine	-6.13	Zalcitabine	-4.99
31	Amantadine	-4.7	Zalcitabine	-6.12	Fosamprenavir	-4.94
32	Zalcitabine	-4.69	Tenofovir	-6.05	Oseltamivir	-4.94
33	Famciclovir	-4.58	Didanosine	-6.03	Saquinavir	-4.88
34	Adefovir	-4.55	Oseltamivir	-5.89	Stavudine	-4.83
35	Penciclovir	-4.44	Idoxuridine	-5.82	Famciclovir	-4.81
36	Acyclovir	-4.23	Tromantadine	-5.76	Adefovir	-4.71
37	Cytarabine	-4.17	Adefovir	-5.45	Zanamivir	-4.59
38	Emtricitabine	-4.12	Lamivudine	-5.44	Acyclovir	-4.21
39	Amprenavir	-3.68	Emtricitabine	-5.31	Penciclovir	-4.17
40	Lopinavir	-3.67	Fosamprenavir	-5.2	Lamivudine	-4.11
41	Oseltamivir	-3.26	Zanamivir	-5.16	Tenofovir	-4.08
42	Telaprevir	-3.1	Trifluridine	-5.16	Trifluridine	-4.07
43	Ritonavir	-3.07	Acyclovir	-5.14	Cytarabine	-3.66
44	Fosamprenavir	-2.47	Cytarabine	-4.69	Atazanavir	-0.56
45	Zanamivir	-2.18	Amantadine	-3.75	Telaprevir	+2.0

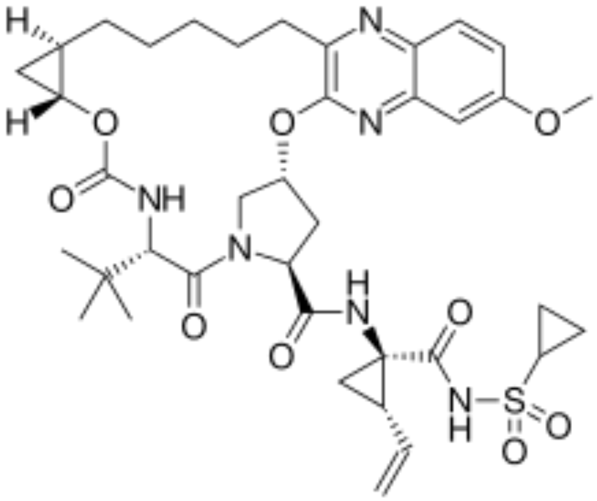

Figure S1: Chemical structure of Grazoprevir

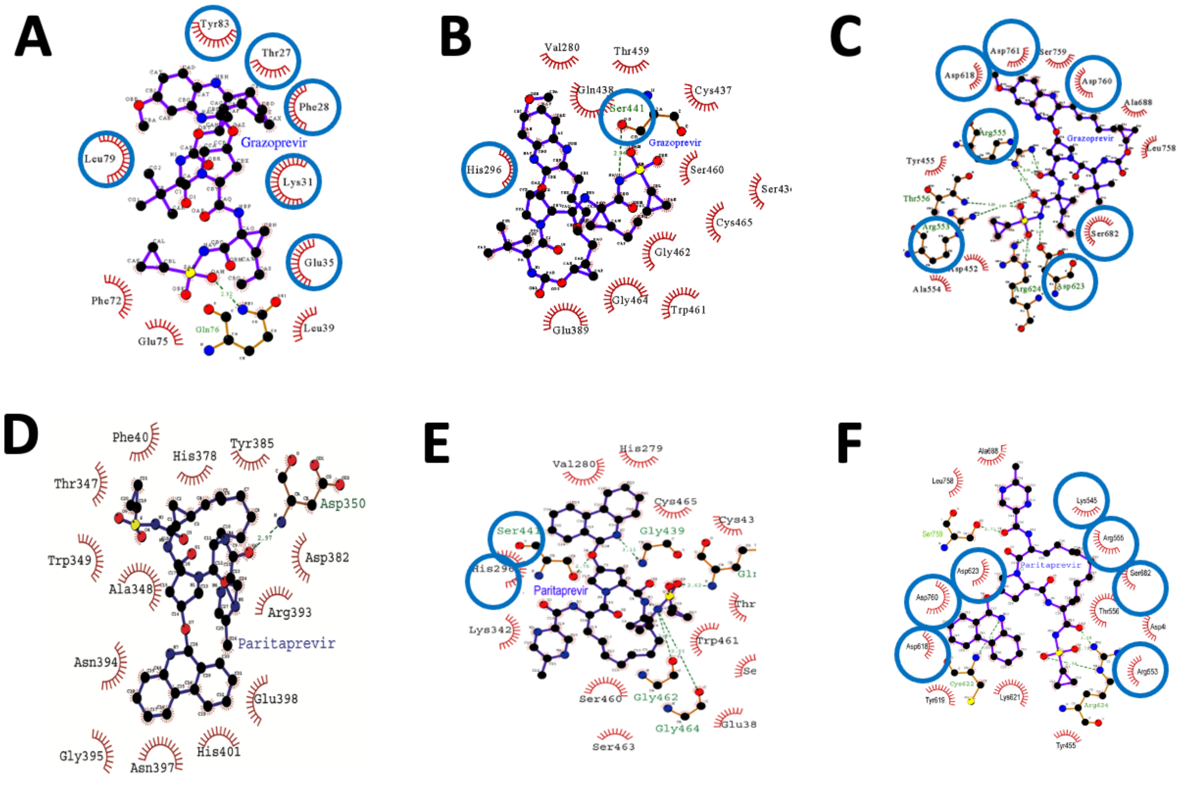

Figure S2: Ligplot analysis displays protein-ligand interactions of (A) ACE2-Grazoprevir, (D) ACE2-Paritaprevir, (B) TMPRSS2-Grazoprevir, (E) TMPRSS2-Paritaprevir, (C) RdRP-Grazoprevir, and (F) RdRP-Paritaprevir complexes showing best fit conformation having highest binding energy selected for each protein, for our further studies, out of all the conformations generated. Hydrogen bond network (green dotted lines) and the other nonbonded interactions for the docked complexes are displayed. All the key key residues interacting with the ligand are highlighted by a blue circle.

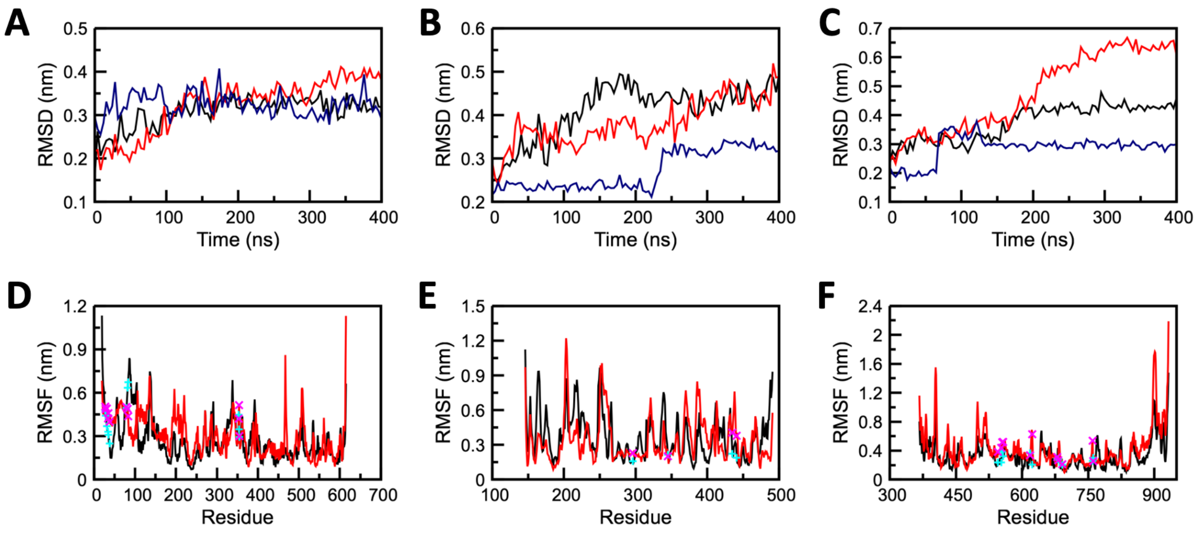

Figure S3: Conformational stability of apo and complex forms of (A, D) ACE2, (B, E) TMPRSS2 and (C, F) RdRP protein. (A, B, C) Backbone-RMSD for protein and all atom RMSD for Grazoprevir and (C, D, E) Residual RMSF. Profile of apo, complex structures and Grazoprevir are displayed in red,

black and blue curves respectively. Locations of key residues are shown by cyan and magenta color symbols for complex and apo states respectively.

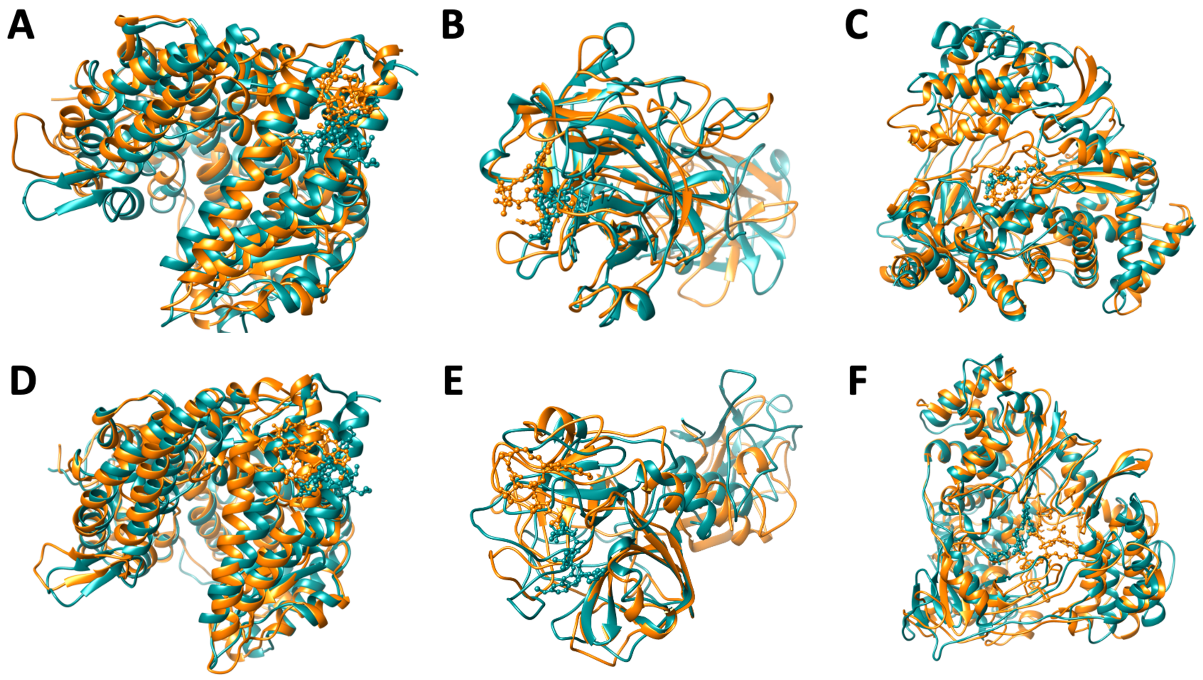

Figure S4: Superimposed structures displaying the binding mode of Grazoprevir before (cyan colour) and after MD simulations (orange colour). (A, D) ACE2-Grazoprevir (B, E) TMPRSS2-Grazoprevir and (C, F) RdRP-Grazoprevir. Structures of A, B, C, and D, E, F from the first and second set of simulations respectively. Protein and Grazoprevir are displayed in the cartoon and ball & stick representations respectively.

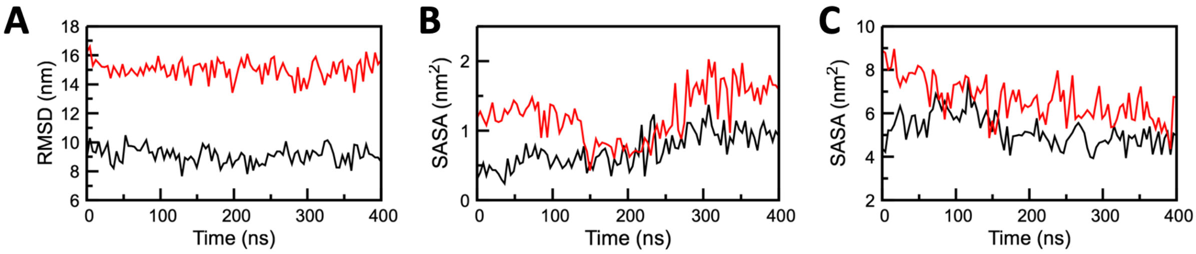

Figure S5: Solvent accessible surface area (SASA) analysis of key residues of ACE2, TMPRSS2 and RdRP during 400 ns MD Simulations; (A) SASA of ACE2 (B)SASA of TMPRSS2 and (C) SASA of RdRP. Complex and apo are displayed by black and red curves respectively.

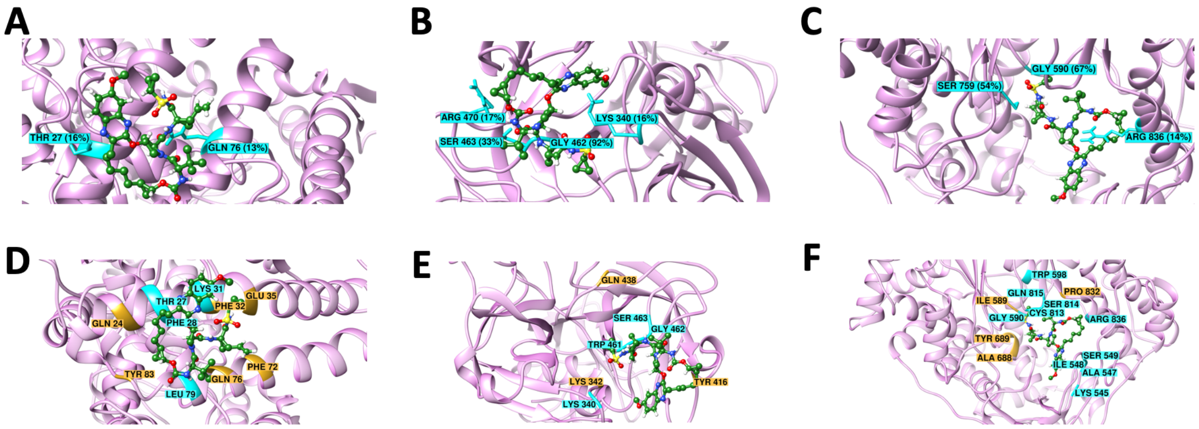

Figure S6: Residues involved in formation of H-bond (A, B, C) and other non-covalent interactions (D, E, F) between (A, D) ACE2 and Grazoprevir (B, E) TMPRSS2 and Grazoprevir (C, F) RdRP and Grazoprevir. Residues forming H-bond that were stable for more than 10% are shown by cyan stick in ribbon diagram with respective stability. Other stable non-covalent interactions having more than 80% stability are shown by cyan ribbon. Contacts having 60-80% stability are shown by orange colour. Grazoprevir is shown in ball and stick representation and carbon, oxygen, sulphur and hydrogen atoms are shown in green red, yellow and white colour respectively.

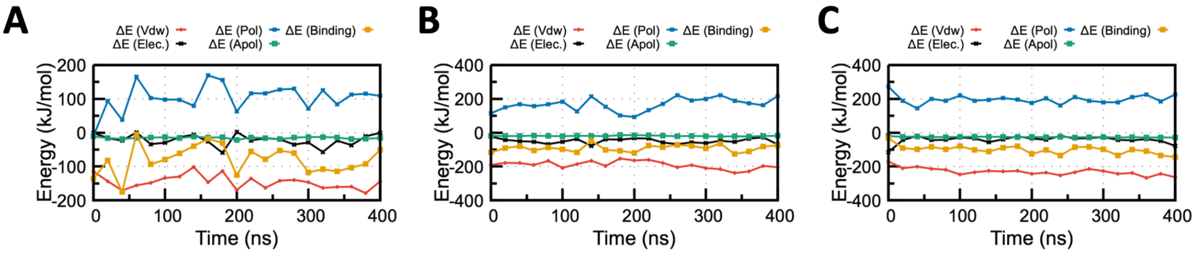

Figure S7: MM-PBSA binding energy profile for the (A) ACE2- Grazoprevir (B) TMPRSS2-Grazoprevir and (C) RdRP-Grazoprevir complex. Contributions from various energy components are also shown for all the three complex. Van der Waals energy (ΔEvdW), Electrostatic energy (ΔEElec), Polar solvation energy (ΔEpol) , Apolar solvation energy (ΔEApol), Total binding energy (ΔEBinding) are displayed by red, black, blue, green and orange curve respectively.

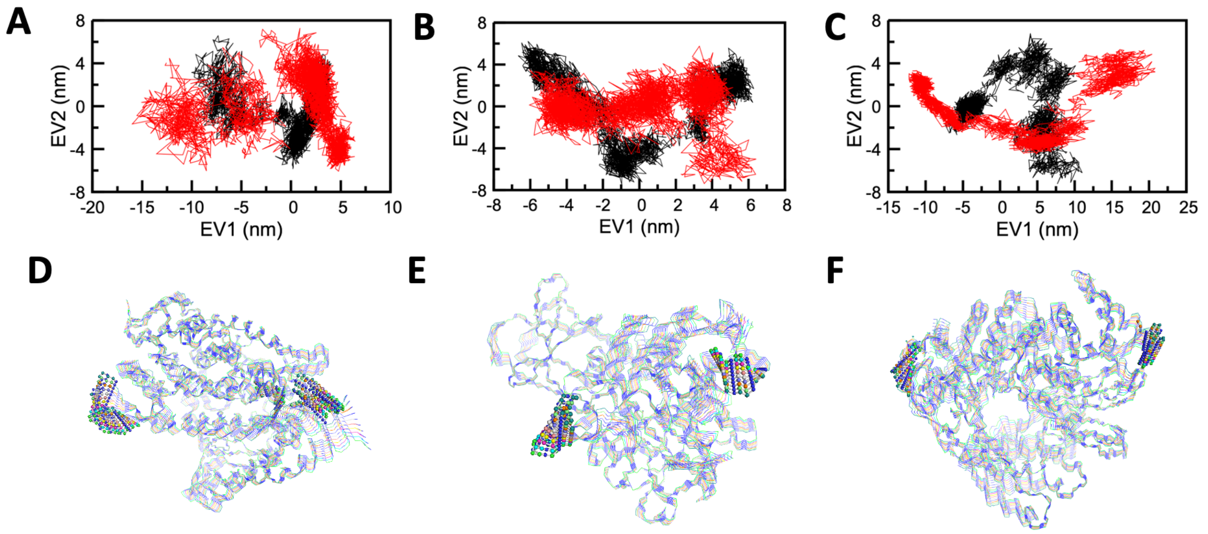

Figure S8: Projection of the motion of the apo and holo forms of ACE2, TMPRSS2, and RdRP in phase space along the first two principal eigenvectors (EV1 and EV2) for (A) ACE2 (B) TMPRSS2 and (C) RdRP. Graphical representation of 10 equally divided structures extracted from dynamic trajectories showing prevalent motions in (D) ACE2 (E) TMRRSS2 and (F) RdRP. Residues found to be highly mobile in comparison to other residues in the targeted proteins depicted in the ball and stick representation.

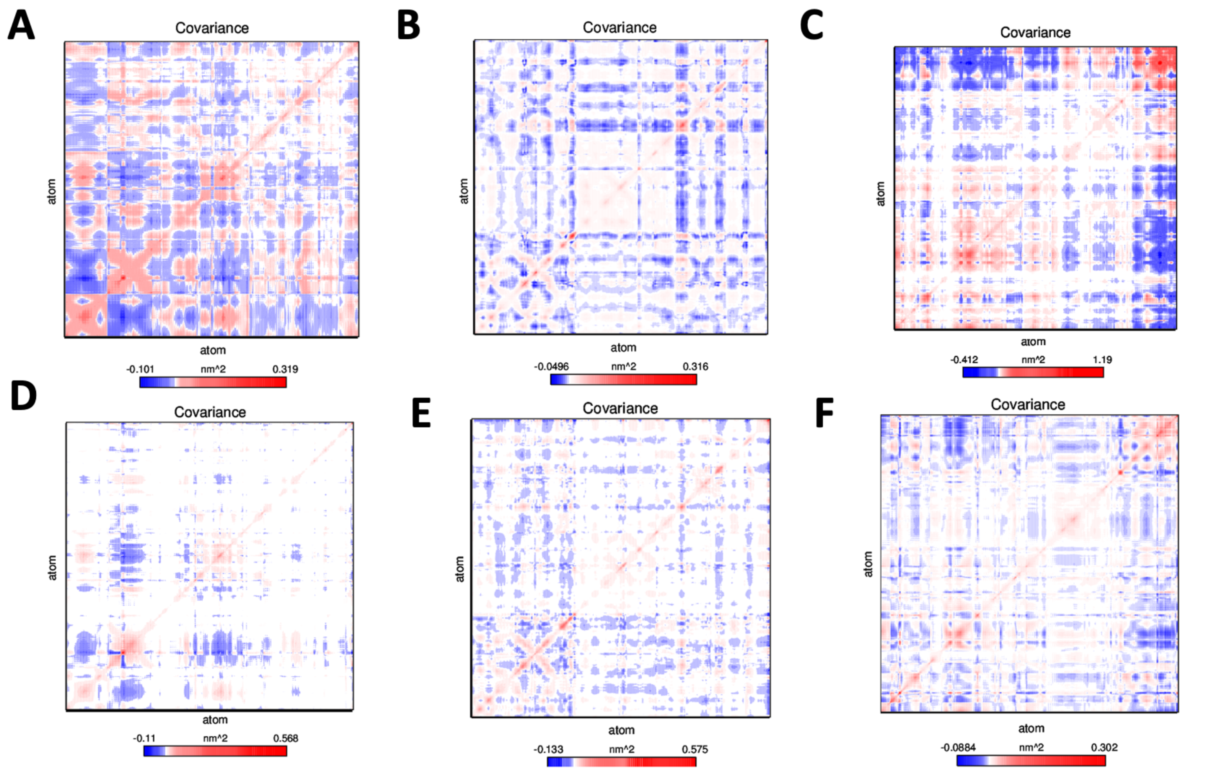

Figure S9: Comparative study of cross-correlation matrices of backbone atoms of (A) ACE2 apo (B) TMPRSS2 apo (C) RdRP apo (D) ACE2-Grazoprevir Complex (E) TMPRSS2-Grazoprevir Complex

(F) RdRP-Grazoprevir Complex during 400 ns MD simulation (set-I). The movement's range is depicted via different colors in the graph panel. Red shades indicate a positive correlation, whereas blue shades indicate an anti- correlation.

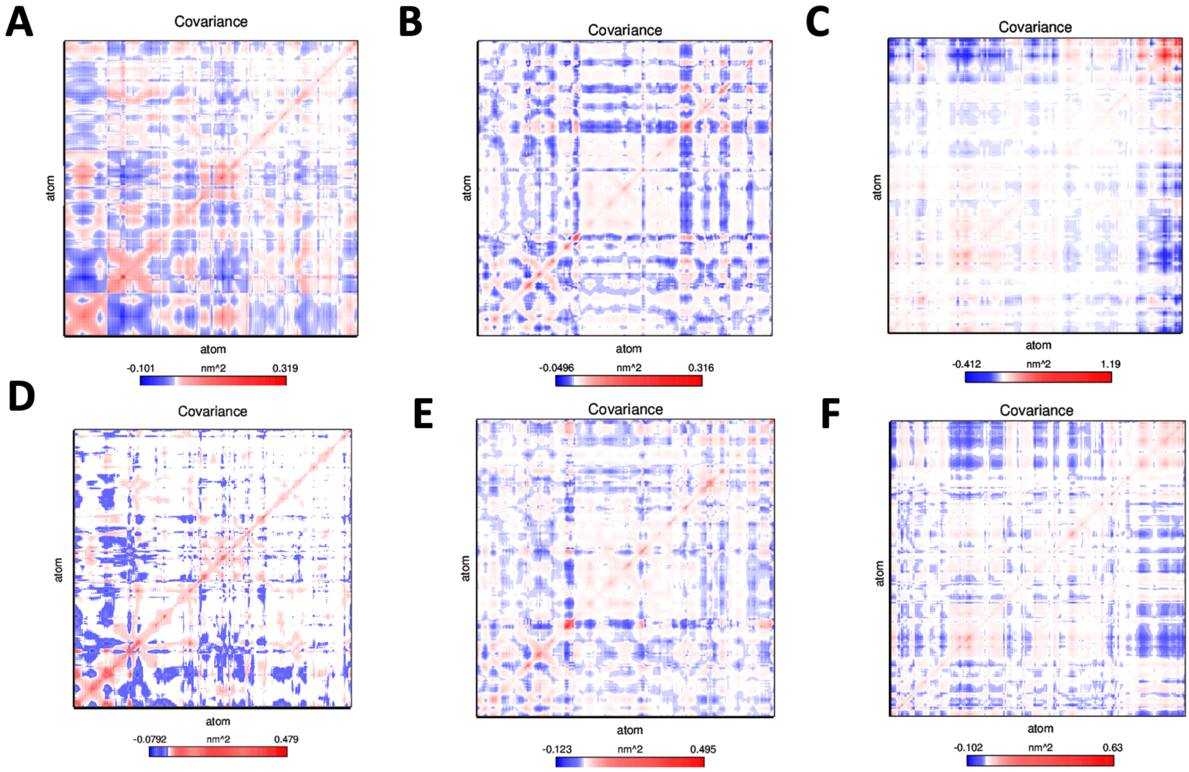

Figure S10: Comparative study of cross-correlation matrices of backbone atoms of (A) ACE2 apo (B) TMPRSS2 apo (C) RdRP apo (D) ACE2-Grazoprevir Complex (E) TMPRSS2-Grazoprevir Complex (F) RdRP-Grazoprevir Complex during 400 ns MD simulation (set-II). The movement's range is depicted via different colors in the graph panel. Red shades indicate a positive correlation, whereas blue shades indicate an anti- correlation.
